# A randomised controlled trial for overweight and obese parents to prevent childhood obesity - Early STOPP (STockholm Obesity Prevention Program)

**DOI:** 10.1186/1471-2458-11-336

**Published:** 2011-05-18

**Authors:** Tanja Sobko, Viktoria Svensson, Anna Ek, Mirjam Ekstedt, Håkan Karlsson, Elin Johansson, Yingting Cao, Maria Hagströmer, Claude Marcus

**Affiliations:** 1 Department of Clinical Science, Intervention and Technology, Division of Paediatrics, Endocrine Research Unit B62, Karolinska Institutet, S-141 86, Stockholm, Sweden; 2 Ersta Sköndal Högskola, Box 111 89, SE-100 61 Stockholm, Sweden; 3 Division of Physiotherapy, Department of Neurobiology, Care Sciences and Society Karolinska Institutet, S-141 86, Stockholm, Sweden

## Abstract

**Background:**

Overweight and obesity have a dramatic negative impact on children's health not only during the childhood but also throughout the adult life. Preventing the development of obesity in children is therefore a world-wide health priority. There is an obvious urge for sustainable and evidenced-based interventions that are suitable for families with young children, especially for families with overweight or obese parents. We have developed a prevention program, Early STOPP, combating multiple obesity-promoting behaviors such unbalanced diet, physical inactivity and disturbed sleeping patterns. We also aim to evaluate the effectiveness of the early childhood obesity prevention in a well-characterized population of overweight or obese parents. This protocol outlines methods for the recruitment phase of the study.

**Design and methods:**

This randomized controlled trial (RCT) targets overweight and/or obese parents with infants, recruited from the Child Health Care Centers (CHCC) within the Stockholm area. The intervention starts when infants are one year of age and continues until they are six and is regularly delivered by a trained coach (dietitian, physiotherapist or a nurse). The key aspects of Early STOPP family intervention are based on Swedish recommendations for CHCC, which include advices on healthy food choices and eating patterns, increasing physical activity/reducing sedentary behavior and regulating sleeping patterns.

**Discussion:**

The Early STOPP trial design addresses weaknesses of previous research by recruiting from a well-characterized population, defining a feasible, theory-based intervention and assessing multiple measurements to validate and interpret the program effectiveness. The early years hold promise as a time in which obesity prevention may be most effective. To our knowledge, this longitudinal RCT is the first attempt to demonstrate whether an early, long-term, targeted health promotion program focusing on healthy eating, physical activity/reduced sedentary behaviors and normalizing sleeping patterns could be effective. If proven so, Early STOPP may protect children from the development of overweight and obesity.

**Trial registration:**

The protocol for this study is registered with the clinical trials registry clinicaltrials.gov, ID: ES-2010)

## Background

Overweight is considered to be a global epidemic and the marked increase in childhood obesity is alarming. Preventing the development of obesity in children is therefore a world-wide health priority [1]. Childhood obesity is multi-factorial and has been recognized as heritable [2]. The odds for a child to become obese as an adult increase about threefold if one of the parents is obese and rise tenfold with two obese parents [3]. To which extent this hereditariness is socio-cultural, genetic or epigenetic is unclear [4].

Gain in body weight and fat mass is a consequence of a positive energy balance over time. Thus, it has been suggested that the rise in the obesity prevalence, even in children, is most likely due to the environmental changes, such as easy access to large portions of energy-dense foods or reduced levels of physical activity (PA), particularly in home environment, or a combination of both [5]. Recent research has demonstrated that additional factors, such as sleep patterns, may also play a role in this negative trend in children [6-8]. In spite of the growing evidence for a relationship between sleep duration and weight gain during infancy [7-10] most family-based interventions for pediatric obesity still focus on eating and PA [11], which calls for multifaceted intervention studies including sleep, especially for very young children.

Although, school-based obesity prevention might be successful [12], the overall effectiveness of intervention programs on obesity prevention has been disappointing, especially when it comes to infants and those in the risk groups to develop obesity. Dietary habits in children [13] are also evident in infants [14], proving that obesity-promoting behaviours learned and supported during the early years may lead to lifestyle behaviours that will track throughout the lifespan. We therefore posit that early childhood provides a unique opportunity to establish lifestyle habits that will promote health and minimize the risk of the development of obesity.

Parental involvement is logically important in weight management in overweight children, yet not much is known about the parental role in preventing childhood overweight in the early life years [15]. Parenting skills are the foundation for successful intervention, since they shape the infant's eating, sleep and PA behaviour. Health promoting information and advices are useless unless the parents cooperate; for example, parents, via their dietary knowledge, parenting style and food environment influence children's emerging food choices [16]. Malfunctioning families may benefit less from the intervention programs if the parenting and family function is not taken into account while designing these programs [11]. This introduces the need for both structured and individually targeted interventions in concordance with the individual parent's learning needs (i.e. knowledge deficits, skills, training needs) [11]. The intervention designed here has therefore taken into consideration that (a) obesity promoting behaviours are established early in life, (b) parents play a crucial role in shaping these behaviours, (c) intervening before these behaviours are established is likely to be effective, and (d) that the individually adjusted coaching is likely to facilitate the uptake of consolidation of health promotion messages.

The aim of this paper is to describe and explain the design and evaluation of the Early STOPP intervention, targeting families where one parent is obese or both parents are overweight. The description of the study protocol follows the checklist of the CONSORT statement for cluster randomized trials [17]

## Design and methods

### Study objectives

The objectives of the Early STOPP randomised controlled trial (RCT) is to study whether a targeted intervention can prevent the development of overweight and obesity among pre-school children in families with either one obese or two overweight parents.

Specifically, this RCT aims

• To compare BMI-sds changes from 1 to 6 years of age in intervention and control families

• To identify modifiable habits that promote wellbeing or contribute to reduced obesity development

• To study whether it is possible to introduce and maintain healthy routines concerning PA, sleep and eating habits in children 1-6 years of age.

• To study the interaction between factors of potential importance for weight increase in this age group such as genetics, sleeping patterns, PA, eating patterns, intestinal bacteria and social family factors as well as how they affect a successful obesity prevention in children during the first 6 years of life.

• To identify and analyse barriers in the implementation of the intervention

### Hypothesis

In comparison to the control group, during the course of intervention, children in the intervention group families are expected: to have reduced BMI gain, to consume more fruits and vegetables and less unhealthy foods such as soft-drinks and energy-dense snack foods, to spent more time being physically active and less time sedentary, specifically TV viewing and computer games, to sleep independently and have healthy sleep habits.

### Overall trial design

**Figure 1:**
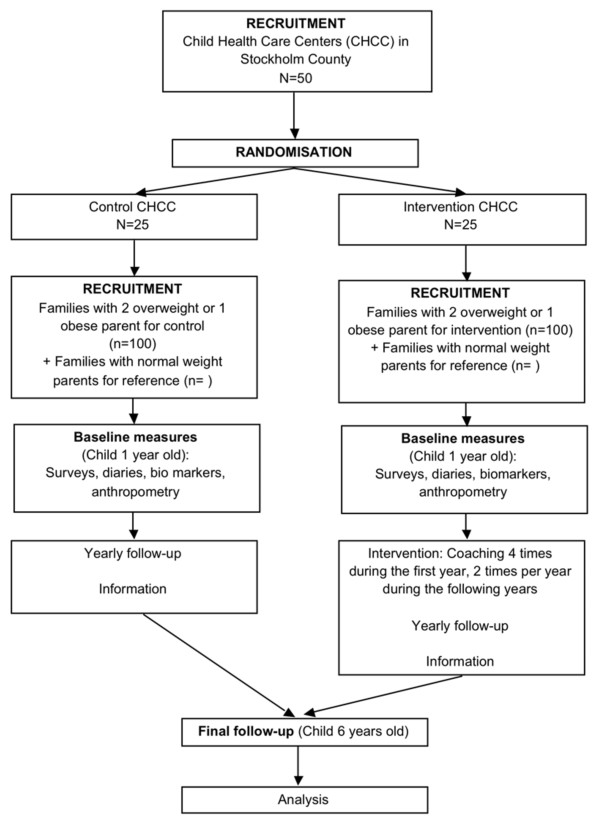
**Diagram of study design and participant flow through the Early STOPP trial**.

The Early STOPP study is a RCT with a longitudinal design targeting overweight and/or obese parents visiting Child Health Care Centres (CHCC) within the Stockholm area, as the units of cluster randomization. The intervention will run from 1 year of age to the 6 year of age (school start). The overall study design is summarized in Figure 1.

### Participants, recruitment and randomization

In total 200 families of different ethnic, socioeconomic and educational background with a child aged 12 month are being recruited at the moment. The included families consist of at least one obese (BMI ≥ 30) or two overweight (BMI ≥ 25) parents. In order to ensure baseline equivalence and minimize selection bias, the overweight and/or obese parents are randomized after recruitment using a computer generated random number schedule developed by a blinded statistician to the Highly Intensive Intervention (HII) and Low Intensive Intervention (LII) groups. The families are informed at baseline on the outcome of randomization prior to the first intervention session. To improve the validity of the measurements of healthy and unhealthy behaviours a Reference group of children (n = 50) with lean parents are being recruited. Representativeness of the trial cohort will continue to be monitored throughout data collection. Health professionals working within the local CHCC are informed about the study through telephone calls, flyers, e-mail bulletins and personal presentations. The parents visiting local CHCC during the routine check up at the age of 8 month are initially approached by the CHCC nurse who gives first brief information about the Early STOPP study and asks if a member of the Early STOPP research team can call and explain more about the study. If so, during the following four months, the Early STOPP team member also answers the questions and if agreed, sends a research information package. The package contains general study information and consent forms.

### Inclusion and exclusion criteria

Parents, at least one obese or two overweight, are eligible to participate if they are able to communicate in Swedish, can freely give informed consent and have a child of one year of age. Infants with chronic health problems that are likely to influence height, weight, levels of PA or eating habits are excluded from this study but are welcome to receive the health promoting information.

### Sample size

The intervention is planned to include 200 families, 100 in each group, providing 80% power at the 5% significance level (double-sided test), considering a 15-20% dropout rate (based on the previous STOPP study). The power calculation is based on detecting a 50% lower prevalence of child overweight and obesity in the intervention group; 20% in the intervention group compared to 40% in the control group.

### Intervention group

The intervention consists of two components, one educational and one individually targeted coaching. The underlying theoretical framework for the intervention is on adult learning theories, academic detailing and coaching [18]. The education and coaching are aimed at raising awareness, increasing parents' knowledge, increasing their self-efficacy to promote parenting behaviour regarding eating, PA and sleep. The multi-disciplinary approach integrates knowledge and methods from medicine, nursing, physiotherapy, dietary or behaviour science. The Early STOPP trial differs from other known prevention programs in the following: a) it starts as early as one year of age, b) aims to effect children's eating, PA and sleep, and c) takes into consideration both parenting skills and parenting style (attitudes that create a certain emotional climate towards eating habits, PA and sleep). The individually adjusted coaching is likely to facilitate the uptake of consolidation of health promotion messages. The key aspects of the Early STOPP program are based on Swedish recommendations for Health Care Centres and include advices on healthy food choices and eating patterns, increasing PA/reducing sedentary behaviour and regulating sleeping patterns.

### Educational component

The educational material (booklets) is developed for correspondent age (1, 2, 3, 4, 5 and 6 years) and is given to the parents/carers to take home during baseline measurements and then during the yearly check up. Advices are based on the latest literature on the subjects of diet, PA and sleeping habits. For example, the sleep part of the intervention methods such as extinction and settled bedtime routines have been tested in treatment of insomnia in childhood, BIC [19,20], as well as sleep and settling behaviors information to parents in the post-natal period has successfully been tested on infants sleep [21].

### Individually targeted coaching component

The coaching is based upon Motivational Interviewing [22] and is also inspired by other theories such Social cognitive theory [23] and the behavioral model "Transtheoretical model of Change" by Prochaska and DiClemente [24]. The coaching is delivered by a trained coach (dietitian, physiotherapist or a nurse) four times the first year and twice a year thereafter. The coach makes an appointment with the family on a time that fits the family. The coaching takes place in the family's home, or if requested, at the clinic. Each coaching session is planned to take around one and a half hour the first visit and one hour the other session. The coaching includes goal setting, report of the current situation, identifying problems, assisting in problem solving; and providing positive encouragement for further development. If the family wishes additional advice between the coaching sessions, they are contacted via telephone and mails, all contacts are documented and stored in the family's files. These ways of communication could provide regular contact with a greater number of families.

### Control group

The control group families receive usual care from their CHCC nurse. In addition, these families will get a general health newsletter at baseline and are thanked yearly with gifts (to a maximum value of €20.00) after completed questionnaires.

### Reference group

The reference group of children with lean parents will receive usual care from their CHCC nurse and are thanked yearly with gifts (to a maximum value of €20.00) after completed questionnaires.

The Regional Ethical Review Board in Stockholm County has reviewed and approved the protocol dnr 2009/217-31.The protocol for this study is registered with the clinical trials registry (clinicaltrials.gov, ID: ES-2010)

## Intervention fidelity and data warehouse

### Quality control

To assure the quality of intervention delivery, all Early STOPP workers, who recruit and administer the baseline questionnaire, have initial training in how to explain the study, how to take consent and how to administer the questionnaire. Personal coaches receive standardized training. A coach's manual contains detailed information specific to each group including the educational material, timing of each session, suggestions and tips to the families. Coaches attend a weekly meeting with the project manager to ensure the protocol adherence. Everyone taking anthropometric measurements, including project workers and clinical nurses is trained in the appropriate technique how to check, calibrate and maintain the necessary equipment. Audio and videotaping of intervention sessions is conducted to assess fidelity of delivery using standardized forms. Using these, the coaching and feedback is provided to the coaches to optimize the intervention delivery.

### Data handling

All data collected according to the agreed Standard Operating Procedures (SOPs) is given a unique study identifier and stored in a data warehouse. This includes data from the clinical analysis of blood samples, data from the baseline questionnaires, and routine clinical data (Table 1). All deviations from the SOPs, such as temperature changes or freeze/thaw episodes, and equipment failures, are recorded. Review of all storage equipment will be done annually. Quality control and maintenance of the data warehouse is the responsibility of the PIs and is controlled by the strict rules of the Biobank laws at Karolinska Institutet.

**Table 1:** Study measures and timing.

	Age (Years)					
	
	1	2	3	4	5	6
**Anthropometry**						
BMI/Waist (C+P)	X	X	X	X	X	X
Blood pressure (C+P)	X	X	X	X	X	X
						
**Biomarkers**						
Blood (C+P)	X		X			X
Feces (C)	X	X	X	X	X	X
Saliva (C+P)	X	X	X	X	X	X
						
**Diet**						
Survey (C+P)	X	X	X	X	X	X
Diary (C+P)	X	X	X	X	X	X
						
**Physical Activity**						
Survey (C)	X	X	X	X	X	X
Accelerometer (C+P)	X	X	X	X	X	X
Hempel Assessment (C)		X		X		X
						
**Sleep**						
Survey (C+P)		X		X		X
Diary (C+P)		X		X		X
Accelerometer (C+P)		X		X		X
						
**Stress**						
Survey (P)		X	X			X

C = Child, P = Parents

## Measures

Primary and secondary outcomes are measured as detailed below.

### Primary and secondary outcomes

Primary outcome is BMI sds-score and the secondary outcomes are PA, sedentary behaviour, motor function/development, sleeping habits, food intake, eating patterns and quality of life. These outcomes require repeated collection due to the rapid changes in height, weight, eating, motor development and PA behaviours in infants. They will therefore be measured at baseline (1 year of age) and yearly thereafter. For children in the intervention group, height, weight and waist circumference are measured every 6 month. Metabolic profile indicators, secondary outcomes, as reflected in biomarkers will be collected at the age of 1, 3 and 6.

Data on demography and socio-economic status is collected by self-report. Parent and infant data is collected by parental/carer self-completed questionnaires and objective measures (Table 1). The questionnaires include already existing validated questionnaires as well as additional questions developed by the Early STOPP project team for topics not reflected in the existing questionnaires. The questionnaires are completed by each parent separately at home before the annual visit. Together these questionnaires take between 30-45 minutes to complete.

### Anthropometry and blood pressure

Trained staff members, using standard procedures and calibrated instruments, collect height, weight and waist-circumference on the infant and parents. Weight is measured with portable scales (Tanita HD-316, Tanita Corp.; Tokyo, Japan) to the nearest 0.1 kg, with shoes and heavy clothing removed. Height is measured to the nearest 0.1 cm using a fixed stadiometer. Based upon height and weight BMI is calculated. Waist circumference is measured right between the lower costal (rib) border and the iliac crest using a nonextensible tape. BMI and waist circumference sds-score will be calculated based upon age- and sex-specific reference values [25,26]. Systolic and diastolic blood pressure (BP) are measured using an automated BP monitor (Dinamap model 8101, Critikon Inc.; Florida, USA) under standard conditions [27]. All of these measurements take place in a private room.

### Dietary intake and eating behaviour

A four-day estimated food diary, covering two weekdays and two weekend days, will assess the children's dietary intake. This diet assessment method is chosen since both qualitative and quantitative aspects of the children's diet intake will be evaluated [28]. The parents' diet quality will be assessed using a semi-quantitative food frequency questionnaire (FFQ) developed and validated by the Swedish National Food Administration. To measure children's eating habits; the Children's Eating Behaviour Questionnaire (CEBQ) will be used [29]. The CEBQ is a multi-dimensional, parent-report questionnaire aims to provide a useful measure of eating styles to study the early precursors of obesity and eating disorders. The CEBQ consists of 35 statements originally derived from interviews with parents about their children's eating behaviour and the literature on the subject. Originally the items covered eight dimensions (factors) of eating style: Food Responsiveness (FR), Emotional Over-Eating (EOE), Enjoyment of Food (EF), Desire to Drink (DD), Satiety Responsiveness (SR), Slowness in Eating (SE), Emotional Under-Eating (EUE) and Food Fussiness (FF).

Outcome measures from the food diary, FFQ and CEBQ will be energy and nutrient intake, meal patterns, times and quantity of vegetable and fruit intake as well snacking patterns.

### Physical Activity, sedentary behaviour and motor function

Parents will report the frequency and duration in health related PA and sedentary behaviour during the previous week using the International Physical Activity Questionnaire (IPAQ), long version [30] at baseline. This is done to be able to compare with national PA data. When the children are 2, 3, 4, 5 and 6 years of age the parents will answer a question about their leisure time PA in the past 12 months [31]. This is done to get a crude measure of the parent's PA levels over a year. Further, the parents will also report the number of hours their child typically spends playing outdoors on weekdays and weekend days. In addition, every year, parents and the child's PA and sedentary behaviour will be assessed objectively using the ActiGraph GT3X+ accelerometer (ActiGraph, LLC, Fort Walton Beach, FL). The ActiGraph has shown high agreement with energy expenditure and measures of time spent in different intensities for adults as well as for children 3 year and up [32-34]. A calibration study on children 2 years of age will be performed within the Early STOPP project. Outcome measures from the accelerometer will be total PA expressed as counts per minute, time spent in different intensities of PA as well as sedentary time. The families will get verbal and easy to read instructions from the research staff on how to wear the accelerometer (around the non-dominant wrist using an elasticated belt). The participants are asked to wear the accelerometer for seven consecutive days, but for the analysis we will include participants that have at least four days of measurements [35].

Motor function of the children will be assessed using the technique described by Hempel [36]. The assessment is age specific and will take place when children are 2 and 4 years of age, respectively. The assessment takes about 30 minutes and will be videotaped to enable analysis afterwards. The examination focuses on motor-functions such as prehension, sitting, crawling, standing and walking behaviours, in a standardized free-field situation. The examination is based mainly on observation of spontaneous motor behaviour since observation is a suitable tool for assessment of qualitative aspects of motor function. The assessment results in three outcome measures; a fluency score, mainly considering fluency of movements (range 0-13); a Clinical classification with four categories ranging from normal to abnormal; a Neurological Optimality Score (range 0-58) [37]. The inter-rater reliability of the Hempel assessment is satisfactory [38].

### Sleeping habits

Assessment of children's and parents' day-to-day sleep pattern for weekdays and weekend at age 2 will be conducted and the ActiGraph GT3X+ accelerometer will be used. The ActiGraph provides an objective recording of sleep duration, sleep efficiency and sleep timing, and is a reasonable tool to evaluate how sleep varies on a day-to-day basis. ActiGraph has shown a good reliability compared with polysomnographic measures of sleep in adults [39,40] and young persons [40,41]. It is possible that the accelerometer does not discriminate entirely between sleep and motionless wakefulness, for example watching TV, which is why sleep diaries are preferred as a complement. For this purpose parents will be asked to complete the brief Infant Sleep Questionnaire (BISQ) [42] to assess children's sleep on a daily basis (7 days). The BISQ has been validated against accelerometry and daily-logs and its sensitivity in documenting expected developmental trends in young children's sleep and the effects of environmental factors have been established [42]. Outcome measures are sleep duration and sleep efficiency (time in bed/sleep time), and settling times. Parents sleep pattern and sleep behaviour will be assessed with the Karolinska sleep/wake diary [43] and parents habitual sleep quality will be assessed with the Karolinska Sleep Quality Index (SQI) [44]. The SQI has been validated against polysomnography and show good correlations with objective sleep parameters [45]. Parenting stress will be assessed by Parenting Stress Index (PSI) [46], the Swedish version.

### Metabolic biomarkers

Fasting blood sample (50 ml for parents and 12 ml for child) is collected by a trained nurse from both parents and the child. The blood sample is analysed for inflammatory markers, cholesterol (total, HDL & LDL), triglycerides, insulin, glucose and hormones (leptin, ghrelin, adiponektin) using standard automated techniques at the KI laboratory. Parents are asked to provide a saliva sample at baseline and at follow-up at 3 and 6 years visits and stored in biobank, this will be used as a source of genomic DNA. Further, nitrate/nitrite levels will be measured in saliva and blood samples and correlated with blood pressure in both parents and their children. Saliva samples will be collected at the same time in tubes containing EDTA, with final concentration of 2 mM and stored at -80°C for later nitrate and nitrite determination. Samples will be analyzed by ion chromatography (ENO 20 Analyzer; Eicom, Kyoto, Japan). To assess the faecal bacterial flora, the microbiological samples will be taken from the stool and analyzed by using a highly parallel 454 Life Sciences GS20 pyrosequencer [47-49].

## Planned data analysis

To achieve a normal distribution the variables will be transformed if needed or non-parametric statistics will be used. Analysis of covariance will be used to test for differences in baseline characteristics between the study arm and adjustment will be made to analyses as required. An estimation of intervention effect on outcome measures will assessed at each follow-up observation (every year post-baseline) on an intention to treat basis. A mixed model will be used to test the average effect of the intervention after taking account of baseline covariates.

## Time plan for the Early STOPP RCT

The study aims to recruit 250 families. Recruitment began in winter 2010 and by Autumn 2011 it is anticipated that all participants will have completed the baseline measurements. The study will continue until children are 6 years of age. It is anticipated that protocols for each phase of follow up of participants in this study will be further adjusted.

## Discussion

To our knowledge, the current study is the first RCT of an early, family-based, lifestyle program for families "at risk" (overweight and obese parents) that will evaluate the effectiveness as well as the sustainability of an individual coaching (i.e. via personal contacts, telephone, and/or electronic mail (email) over a five-year period.

Early years of life is a time in which obesity prevention may be most effective. To our knowledge this will be the first longitudinal randomized trial to demonstrate if an early individually adapted health promotion program delivered to overweight or obese parents promotes healthy eating, PA and balanced sleeping habits. If proven to be effective, Early STOPP may protect children from the development of obesity and associated socio-economic costs.

## Competing interests

The authors declare that they have no competing interests.

## Authors' contributions

All authors were involved in the development and application of the protocol of this study. The contributions of other members of the Early STOPP study team are gratefully acknowledged and listed below.

CM (Principal investigator) and TS were responsible for identifying overall research questions, design of the study, obtaining ethics approval, the acquisition of funding, as well as overseeing the study implementation. TS took the lead in writing and finalizing the design of this study, based on the overall concept and design primarily outlined by CM. VS, ME, MH has provided expertise for the Early STOPP program with particular emphasis on design of dietary habits, sleep, physical activity and statistical analyses. AE, EJ, HK and YC contributed with questionnaires, protocols, tables and figures both in the study and in this manuscript. All authors read and approved the final manuscript.

## Pre-publication history

The pre-publication history for this paper can be accessed here:

http://www.biomedcentral.com/1471-2458/11/336/prepub
